# Prevalence of anemia in patients with chronic kidney disease attended at a nephrology outpatient clinic and its association with the outcome of renal replacement therapy over five years: a retrospective cohort study, Municipality of São Paulo, 2010-2018

**DOI:** 10.1590/S2237-96222026v35e20250206.en

**Published:** 2026-03-09

**Authors:** Andre Kiyoshi Miyahara, Pedro Henrique Moretti Pepato, Júlia Ferreira Rocha, Alexandre Vizzuso de Oliveira, João Vitor Bozza Maia, Vinicius Diniz, Bruno Pellozo Cerqueira, Maria Amelia Aguiar Hazin, Maria Eugenia Fernandes Canziani, Miguel Angelo Goes

**Affiliations:** ¹ Universidade Federal de São Paulo, São Paulo, SP, Brazil

**Keywords:** Renal Insufficiency, Chronic, Anemia, Primary Health Care, Prevalence, Cohort Studies., Insuficiencia Renal Crónica, Anemia, Atención Primaria de Salud, Prevalencia, Estudios de Cohortes.

## Abstract

**Objective:**

To evaluate the prevalence of anemia in patients with chronic kidney disease and its association with the need for renal replacement therapy for five years in a specialized outpatient clinic.

**Methods:**

Retrospective cohort study that included patients admitted to a nephrology outpatient clinic between 2010 and 2018 in the municipality of São Paulo. Demographic, clinical, and laboratory data were analyzed using frequencies and absolute values. Anemia was defined as a hemoglobin concentration <13 g/dL for men and <12 g/dL for women. Clinical data and the need for renal replacement therapy over five years between patients with and without anemia at admission were compared. For statistical analyses, Pearson’s chi-square and Student’s t or Mann-Whitney *U* tests, Kaplan-Meier curve with log-rank test, and survival analysis using Cox regression were used.

**Results:**

The study included 534 patients, 41.8% of whom were diagnosed with anemia at admission. Hypertension (35.2%) and diabetes (27.5%) were the leading causes of chronic kidney disease. In five years, 4.1% progressed to renal replacement therapy. Anemia at admission was associated with a higher risk of the outcome (hazard ratio - HR 3.24; 95% confidence interval-95%CI 1.38; 7.61). Hemoglobin level was an independent predictor of outcome (HR 0.766; 95%CI 0.593; 0.989).

**Conclusion:**

A high prevalence of anemia in chronic kidney disease was found in patients from Primary Health Care. Hemoglobin level was an independent variable associated with the outcome.

## Resumen

**Objetivo:**

Evaluar la prevalencia de anemia en pacientes con enfermedad renal crónica (ERC) y su asociación con la necesidad de terapia de reemplazo renal (TRR) durante cinco años en una consulta especializada. ).

**Métodos:**

Estudio de cohorte retrospectivo que incluyó pacientes ingresados en una consulta de nefrología entre 2010 y 2018 en São Paulo, Brasil. Se analizaron datos demográficos, clínicos y laboratoriales mediante frecuencias y valores absolutos. La anemia se definió como concentración de hemoglobina <13 g/dL para hombres y <12 g/dL para mujeres. Se compararon los datos clínicos y la necesidad de TRR a lo largo de cinco años entre pacientes con y sin anemia en el ingreso. Para el análisis estadístico se utilizaron la prueba de chi-cuadrado de Pearson, la prueba t de Student o U de Mann-Whitney, curvas de Kaplan-Meier con prueba de log-rank y análisis de supervivencia mediante regresión de Cox.

**Resultados:**

El estudio incluyó a 534 pacientes, de los cuales el 41,8% fueron diagnosticados con anemia en el ingreso. Las principales etiologías de la ERC fueron hipertensión arterial (35,2%) y diabetes mellitus (27,5%). A cinco años, el 4,11% de los pacientes evolucionaron hacia TRR. La anemia en el ingreso se asoció con un mayor riesgo del desenlace (hazard ratio 3,24; intervalo de confianza 95% 1,38-7,61). El nivel de hemoglobina fue un predictor independiente del desenlace (HR 0,766; IC 95% 0,593-0,989).

**Conclusión:**

Se encontró una alta prevalencia de anemia en pacientes con enfermedad renal crónica provenientes de Atención Primaria. El nivel de hemoglobina fue una variable independiente asociada al desenlace.

**Ethical aspects**

This research respected ethical principles, having obtained the following approval

Research ethics committee: Universidade Federal de São Paulo

Opinion number: 3,200,969

Approval date: 15/3/2019

Certificate of submission for ethical appraisal: 08636819.8.0000.5505/2019

Informed consent record: Exempt.

## Introduction

Chronic kidney disease is a global public health problem, characterized by morphofunctional changes in the kidneys that persist for more than three months. This condition leads to metabolic and functional complications and may result in adverse clinical outcomes, such as the need for renal replacement therapy, increased cardiovascular risk, bone fragility, anemia, and increased mortality ^1^. Chronic kidney disease is classified based on its etiology, estimated glomerular filtration rate, and the presence of albuminuria ^1^. The main causes of the disease are diabetes and hypertension, which reinforces the importance of preventive management in Primary Health Care. The need for renal replacement therapy arises as chronic kidney disease progresses. Such therapy is indicated when the estimated glomerular filtration rate is less than 15 ml/min/1.73 m2, associated with uremic syndrome, acid-base balance imbalance, difficulty maintaining euvolemia, or blood pressure alterations ^1^. Anemia is a frequent complication in chronic kidney disease, resulting from erythropoietin deficiency and worsened by factors such as nutritional deficiencies and inflammation ^2^ ^,^ ^3^ ^,^ ^4^ ^,^ ^5^ ^,^ ^6^. The inflammatory state associated with chronic kidney disease impairs the action of erythropoietin, reduces intestinal iron absorption, and hinders the mobilization of iron stores due to increased hepatic production of hepcidin ^2^ ^,^ ^3^ ^,^ ^4^. Anemia becomes clinically evident when the estimated glomerular filtration rate falls below 30 ml/min/1.73 m2, intensifying cardiovascular complications and contributing to increased mortality ^6^ ^,^ ^7^.

This study aimed to assess the prevalence of anemia in patients with chronic kidney disease referred from Primary Health Care for conservative treatment at a specialized nephrology unit in the municipality of São Paulo. The study also aimed to analyze the association between anemia and the need for renal replacement therapy over five years. This study aimed to better understand the profile of anemia associated with chronic kidney disease in the Brazilian context, thereby contributing to bridging the gap in epidemiological studies on this condition in the country.

## Methods

Design and setting

This was a retrospective cohort study based on the analysis of medical records of patients admitted to the outpatient service between 2010 and 2018, at a University Medical Service specialized in chronic kidney disease, part of the Nephrology Department of the Escola Paulista de Medicina/Federal University of São Paulo (*Universidade Federal de São Paulo*, UNIFESP). The objective was to evaluate the presence of anemia at admission to the service and compare it between two groups: (i) patients with anemia and (ii) patients without anemia. Patients were monitored for five years to assess the necessity of renal replacement therapy. 

Data sources, measurement, and variables

Clinical and laboratory data were collected from the physical and electronic medical records systems, which included demographic information (sex, age, anthropometric measurements, comorbidities), etiology and stage of chronic kidney disease, as well as medications in use. The laboratory tests evaluated included complete blood count, venous blood gas analysis, lipid profile, glycemic profile, iron profile, creatinine, urea, sodium, potassium, phosphorus, parathyroid hormone, total calcium, and urinalysis. Anemia was defined according to World Health Organization (WHO) criteria: hemoglobin <13.0 g/dL for men and <12.0 g/dL for women ^8^. 

The diagnosis of chronic kidney disease was based on the KDIGO 2024 for chronic kidney disease ^1^. According to the guideline, chronic kidney disease is a morphofunctional alteration lasting three months or more, which can be identified by the presence of one or more markers of kidney damage: albuminuria (≥30 mg/g), abnormal urinary sediments, persistent hematuria, electrolyte imbalances due to tubular abnormalities, pathologies detected by histology, structural abnormalities detected by imaging, or a history of kidney transplantation. Renal function <60 ml/min per 1.73 m2 lasting more than three months is considered chronic kidney disease, which is classified into:

G1 ≥90 ml/min per 1.73 m2; G2 60-89 ml/min per 1.73 m2; G3a 45-59 ml/min per 1.73 m2; G3b 30-44 ml/min per 1.73 m2; G4 15-29 ml/min per 1.73 m2; G5 <15 ml/min per 1.73 m^2 (^ ^1^.

Participants

The inclusion criteria were: patients diagnosed with chronic kidney disease admitted between 2010 and 2018 who had complete laboratory tests, including blood count, urea, creatinine, sodium, and potassium. The exclusion criteria were: age under 18 years; anemia from other etiologies; initiation of renal replacement therapy or kidney transplantation within 90 days after inclusion; absence of an adequate diagnosis of chronic kidney disease; and death occurring within the first 90 days after inclusion.

Anemia from other etiologies was diagnosed based on clinical history and laboratory tests, such as elevated HDL, Coombs test, and haptoglobin. An expert and trained medical professional evaluated each patient. The excluded patients were not admitted to the outpatient clinic, and there was direct follow-up in other specialties, such as hematology and rheumatology. Thus, in the results, patients from other specialties with anemia of other etiologies who were being followed up through nephrology team consultations were not included. 

The absence of adequate diagnosis of chronic kidney disease was defined as failure to meet one or more established inclusion criteria.

The etiological diagnosis of chronic kidney disease was based on clinical history and analysis of comorbidities, such as hypertension, diabetes, and autosomal dominant polycystic kidney disease. Biopsy was limited to cases with indications for renal biopsy, such as acute nephritic syndrome, nephrotic syndrome, renal failure of unclear cause, and rapidLy progressive glomerulonephritis.

Patients were initially stratified based on the presence or absence of anemia at the beginning of outpatient follow-up. The outcome analyzed was the need for renal replacement therapy within five years of admission. A second analysis was conducted to identify variables associated with progression to renal replacement therapy during this period, as well as the need for erythropoietin use within two years after admission. 

Treatment with erythropoietin followed the Clinical Protocols and Therapeutic Guidelines for anemia in chronic kidney disease from the Ministry of Health, indicating its use when hemoglobin is <10 g/dL ^9^.

Bias control

The single-center design, although facilitating the control of operational and logistical variables, may limit the external validity of the results. This occurs because the population characteristics, care practices, and local infrastructure may not reflect the reality of other centers, introducing selection bias and compromising the study’s external validity.

Study size

The study included all patients admitted to the outpatient clinic who presented complete clinical and laboratory data adequate to meet the inclusion criteria, totaling 534 patients.

Statistical methods

The level of statistical significance was set at a p-value<0.05. Statistical analyses were performed using SPSS Statistics Version 21 software (IBM, Armonk, New York, USA). Categorical variables were expressed as absolute frequencies and percentages, and compared using Pearson’s chi-square test. Quantitative variables were compared between groups using Student’s t-test or Mann-Whitney *U* test, depending on the results of the Kolmogorov-Smirnov normality test. A Kaplan-Meier analysis with the log-rank test was performed to compare renal survival between groups with and without anemia at admission. Renal survival was defined as the absence of the need for renal replacement therapy or kidney transplantation within five years.

Cox regression was used to identify variables associated with the outcome, adjusting for potential confounding factors. The variables included in the regression model were statistically significant and showed no multicollinearity, as assessed by the variance inflation factor diagnostic. The analysis included estimated glomerular filtration rate, total cholesterol, hemoglobin, age, and low-density lipoprotein. 

## Results

At the initial phase of the study, 889 medical records of patients beginning outpatient follow-up for chronic kidney disease at the nephrology service were collected. Next, 355 patients were excluded for the following reasons: being under 18 years old (n=18); having started renal replacement therapy (n=10) or died (n=1) within 90 days after beginning outpatient follow-up; and lacking sufficient examinations for the current study (n=326). 

**Figure 1.**
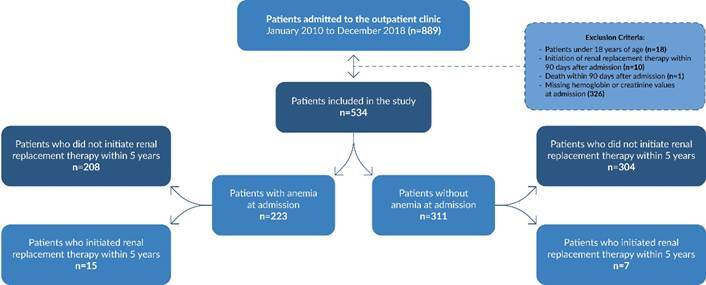
Patients with chronic kidney disease admitted to the outpatient clinic specializing in nephrology and who were included in the study. São Paulo, 2010-2018

The study included 534 outpatients (Figure 1), with a mean age of 69.4 years. Of these patients, 295 (55.2%) were male. The main etiologies of chronic kidney disease in the admitted patients were hypertension (35.2%) and diabetes (27.5%), followed by tubulointerstitial nephritis (12.0%), chronic glomerulonephritis (7.0%), and autosomal dominant polycystic kidney disease (4.0%). The first analysis stratified eligible participants by anemia status at the beginning of outpatient follow-up. It was observed that 223 patients (41.8%) had anemia.

Early stages of chronic kidney disease were more prevalent in patients without anemia than in those with anemia (p-value<0.001). Previous use of iron and vitamin B12 supplementation was more frequent in the group with anemia. There was no statistically significant difference between the groups regarding the use of hypoglycemic agents, vitamin D, and antihypertensives such as angiotensin-converting enzyme inhibitors and angiotensin receptor blockers (Table 1). Of the patients admitted with anemia, 13.9% required recombinant human erythropoietin within two years, compared to 1.6% in the group without anemia (p-value<0.001). 

**Table 1.** Comparison between groups of patients with chronic kidney disease admitted to the outpatient clinic with and without anemia. São Paulo, 2010-2018 (n=534)

	Without anemia (n=311)	Anemia (n=223)	
	n (%)	n (%)	p-value
Male	177 (56.9)	118 (52.9)	0.359
Chronic kidney disease stage			<0.001
1	2 (0.6)	0 (0.0)	
2	14 (4.5)	9 (4.0)	
3A	69 (22.2)	13 (5.8)	
3B	136 (43.7)	74 (33.2)	
4	85 (27.3)	110 (49.3)	
5	5 (1.6)	17 (7.6)	
Medicines in use^a^			
Iron supplementation	6 (2.4)	18 (9.4)	0.001
Vitamin B12	13 (5.1)	21 (11.0)	0.022
Erythropoietin in two years	5 (1.6)	30 (13.9)	<0.001

^a^ Only available to 444 patients.

Patients in the anemia group who began outpatient follow-up had lower estimated glomerular filtration rate, serum bicarbonate, hemoglobin concentration, hematocrit, mean corpuscular hemoglobin concentration, and serum iron levels. In contrast, patients in this group showed higher RDW, potassium, phosphorus, and intact parathyroid hormone levels (Table 2).

**Table 2.** Comparison between groups of patients with chronic kidney disease admitted to the outpatient clinic with and without anemia. São Paulo, 2010-2018 (n=534)

	Without anemia (n=311)	Anemia (n=223)	p-value
	Median (IQR)	Median (IQR)	
Body Mass Index (kg/m^2^)	27.2 (23.9-31.1)	26.3 (23.9-29.7)	0.251^a^
Estimated glomerular filtration rate (ml/min/1,73m²)	35.6 (28.5-45.7)	27.5 (20.3-36.9)	<0.001^a^
Hemoglobin (g/dL)	13.9 (13.1-14.9)	11.2 (10.4-11.8)	<0.001
RDW ^b^ (%)	11.8 (11.3-12.6)	12.3 (11.3-13.2)	0.012^a^
Mean corpuscular hemoglobin (pg)	30.1 (28.5-31.1)	29.5 (27.8-30.9)	0.037^a^
Venous blood gas pH ^c^	7.31 (7.27-7.34)	7.30 (7.25-7.34)	0.064^a^
Bicarbonate (mEq/l)	26.6 (24.1-29.1)	25.4 (22.0-27.8)	<0.001
Iron (mcg/dL)	79.5 (67.0-100.2)	65.5 (50.5-88.2)	0.002
Ferritin (ng/ml)	138.5 (75.1-254.3)	188.5 (91.0-299.5)	0.072^a^
Transferrin saturation (%)	27.0 (22.0-35.0)	25.0 (18.5-33.4)	0.328
Phosphorus (mg/dL)	3.4 (3.0-3.8)	3.8 (3.5-4.3)	<0.001
Sodium (mEq/l)	139 (138-141)	139 (138-142)	0.637^a^
Potassium (mEq/l)	4.7 (4.4-5.0)	4.9 (4.5-5.3)	0.001
PTH (pg/ml) ^d^	99.5 (62.7-156.7)	132.0 (82.0-213.0)	0.001^a^
Total cholesterol (mg/dL)	181 (155-210)	175 (143-207)	0.414

^a^ Variables with non-normal distribution; ^b^ RDW: *red cell distribution width*; ^c^ pH: hydrogen ion potential; ^d^ PTH: parathyroid hormone.

In the second analysis, patients were stratified based on the outcome of requiring renal replacement therapy within five years after starting outpatient follow-up. Baseline clinical data were compared between these groups. Of the 534 patients in the study, 22 (4.1%) progressed to renal replacement therapy within five years, and all were referred to a hemodialysis program. Patients who progressed to dialysis were younger (54.5 [48.0-67.3] versus 65.0 [56.0-73.8] years; p-value=0.030), had a lower estimated glomerular filtration rate (27.0 [15.4-32.0] vs. 32.6 [25.1-42.2] ml/min per 1.73 m^2^; p-value=0.011), higher total cholesterol levels (194.5 [161.8-243.5] vs. 179.0 [151.0-207.8] mg/dL; p-value=0.004) and low-density lipoprotein levels (120.5 [92.8-154.0] vs. 102.0 [80.0-127.0] mg/dL; p-value=0.004), as well as lower hemoglobin concentrations (11.9 [10.5-12.9] vs. 12.8 [11.4-14.0] g/dL; p-value=0.032) at admission (Table 3). 

**Table 3.** Comparison between patients who progressed to renal replacement therapy within five years and those who did not need it. São Paulo, 2010-2018 (n=534)

Variable	No dialysis (n=512)	Dialysis (n=22)	p-value
	n (%)	n (%)	
Male	280 (54.7)	15 (68.2)	0.213
Anemia at admission	208 (40.6)	15 (68.2)	0.011
Etiology of chronic kidney disease			0.012
Diabetes	137 (26.8)	10 (45.4)	
Hypertension	185 (36.1)	3 (13.6)	
Chronic glomerulonephritis	34 (6.6)	5 (22.7)	
Chronic interstitial nephritis	64 (12.5)	2 (9.1%)	
Indeterminate	70 (13.7)	2 (9.1%)	
Autosomal dominant polycystic kidney disease	22 (4.3)	0 (0.0)	
Admission medications^a^			
Angiotensin-converting enzyme inhibitor	155 (36.6)	6 (28.6)	0.457
Angiotensin receptor blocker	117 (27.6)	9 (42.9)	0.130
Loop diuretics	165 (38.9)	14 (66.7)	0.011
Calcium channel blocker	148 (34.9)	12 (57.1)	0.038
Iron supplementation	20 (4.7)	4 (19.1)	0.005
Erythropoietin use within two years	28 (5.5)	8 (36.4)	<0.001

^a^ Only available to 444 patients.

In the dialysis group, a higher prevalence of diabetes as the etiology of chronic kidney disease was observed (45.4% vs. 26.8%; p-value=0.012), as well as a higher frequency of anemia (68.2% vs. 40.6%; p-value=0.011). Of the 22 patients who required dialysis, 36.4% used erythropoietin at some point during the first two years of follow-up, compared to 5.5% of those who did not progress to renal replacement therapy (p-value<0.001). Patients who required renal replacement therapy had higher use of loop diuretics, dihydropyridine calcium channel blockers, and iron supplementation (Table 3). 

**Figure 2:**
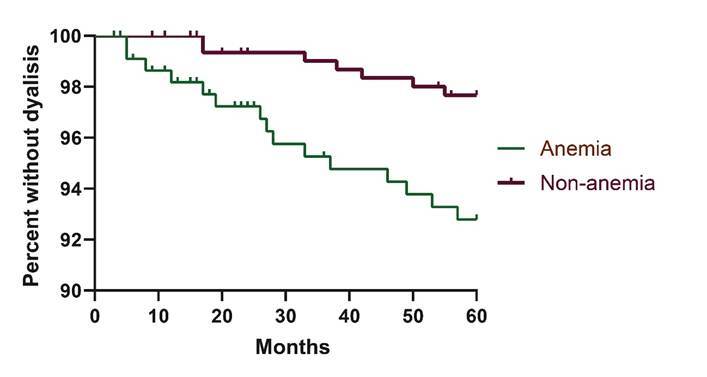
Hazard ratio (HR) and 95% confidence interval (95%CI) for the need for renal replacement therapy within five years of follow-up of patients with and without anemia. São Paulo, 2010-2018 (n=534)

The Kaplan-Meier curve showed that patients with chronic kidney disease and anemia had a higher need for hemodialysis over five years of follow-up (hazard ratio - HR 3.24; 95% confidence interval - 95%CI 1.38; 7.61; p-value<0.001), with 44 patients lost to follow-up (Figure 2). In the Cox regression analysis, estimated glomerular filtration rate (HR 0.977; 95%CI 0.940; 1.017; p-value 0.259), total cholesterol levels (HR 1.009; 95%CI 0.995; 1.024; p-value 0.211), and low-density lipoprotein (HR 1.004; 95%CI 0.986; 1.023; p-value 0.643) did not show statistical significance. Age (HR 0.973; 95%CI 0.948; 0.998; p-value 0.036) and hemoglobin (HR 0.766; 95%CI 0.593; 0.989; p-value 0.041) were identified as significant independent predictors of the outcome.

In the anemia group, one patient (0.4%) died within five years after the start of follow-up. This death occurred three years after enrollment in the outpatient service. No deaths were recorded in the group without anemia during the five-year follow-up. 

## Discussion

The main finding of this study was the frequent presence of anemia in patients with chronic kidney disease initiating follow-up in a specialized outpatient clinic within the Brazilian National Health System (*Sistema Único de Saúde*, SUS). It was identified that 41.8% of the enrolled outpatients presented anemia at admission. Health centers referred these patients through the Health Services Supply Regulation Center of the State of São Paulo (*Central de Regulação de Ofertas de Serviços de Saúde do Estado de São Paulo*, CROSS), which directs referred patients based on prognosis and urgency for service access, considering available schedules and territorial division.

This alarming rate of anemia is not only significant but also far exceeds the prevalence observed in populations with chronic kidney disease in other countries, where anemia is often identified in the early stages of the disease process ^2^ ^,^ ^10^ ^,^ ^11^ ^,^ ^12^. Comprehensive assessments and preventive monitoring are usually performed only in the more advanced stages of chronic kidney disease, which is concerning given the well-documented effects of anemia on health outcomes in patients with chronic kidney disease.

Anemia in chronic kidney disease primarily results from erythropoietin deficiency and is influenced by other factors that worsen as the disease progresses. Reduced erythropoietin production, combined with vitamin deficiencies, inflammation, and altered iron metabolism, impairs red blood cell production. Infections and the accumulation of uremic solutes can exacerbate the inflammatory response or result from comorbidities and underlying health conditions ^2^ ^,^ ^3^ ^,^ ^4^ ^,^ ^5^ ^,^ ^1^ ^,^ ^14^.

The decrease in hemoglobin concentration negatively affected the progression of chronic kidney disease in 265 patients with type 2 diabetes (HR 0.65; 95%CI 0.48; 0.88; p-value 0.005). Lower hemoglobin levels (<13.3 g/dL) were an independent predictor of kidney damage ^15^. The presence of anemia is associated with lower estimated glomerular filtration rate values, regardLess of the cause-and-effect relationship ^4^ ^,^ ^5^ ^,^ ^6^ ^,^ ^12^ ^,^ ^13^. This study aligns with findings in the international literature, which report a higher prevalence of anemia in the advanced stages of chronic kidney disease, suggesting the robustness of the medical-scientific knowledge regarding this condition ^16^ ^,^ ^17^. Even with well-established literature on the consequences of renal anemia, patients with chronic kidney disease and anemia without appropriate therapeutic intervention were still found, especially among non-dialysis chronic kidney disease patients.

The presence of anemia has been associated with a faster rate of progression of chronic kidney disease ^18^. Therefore, this should be one of the key focuses in the recognition and monitoring of the disease. The poor prognosis of outpatients and adverse outcomes may be associated with the concomitant presence of anemia and reduced estimated glomerular filtration rate, such as ischemic cardiovascular events or the need for renal replacement therapy ^19^ ^,^ ^20^. These outcomes carry considerable morbidity and mortality and require medical intervention, leading to increased costs for both public and private health centers. Based on this study and the established literature, the results suggest that general practitioners can monitor anemia from the early stages of chronic kidney disease to mitigate progression to renal replacement therapy, reduce morbidity and mortality, and lower per-patient costs.

The presence of anemia was also associated with higher serum levels of phosphorus and parathyroid hormone (PTH), factors related to mineral and bone disorder-common in the advanced stages of chronic kidney disease-a condition linked to increased morbidity and mortality in this disease 2^1^. The group of patients with anemia showed lower bicarbonate levels, accompanied by a tendency toward lower venous blood gas pH values, which occurs due to changes in renal bicarbonate production and reabsorption, as well as hydrogen ion excretion ^22^. It is estimated that metabolic acidosis is present in approximately 15.0% of patients with chronic kidney disease, with a 40.0% higher risk in anemic patients with chronic kidney disease ^23^. Prioritizing anemia management is essential to improve overall health and reduce complications in patients with chronic kidney disease.

Observations were made regarding the need for renal replacement therapy within five years after the start of specialized outpatient follow-up for chronic kidney disease. A higher frequency of chronic kidney disease etiology due to diabetes (45.5%) than hypertension (13.4%) was observed in patients who progressed to renal replacement therapy. This finding differs from Brazilian literature data, which report that 33.0% of end-stage renal disease cases were attributed to hypertension and 32.0% to diabetes ^24^. However, these findings are consistent with those from other countries, where diabetes is the leading cause of chronic kidney disease progressing to the need for renal replacement therapy ^25^ ^,^ ^26^. Dialysis patients also showed more alterations in their lipid profile, with higher levels of total cholesterol and low-density lipoprotein. Patients with dyslipidemia tend to progress to chronic kidney disease with a worse prognosis and significant cardiovascular risk, requiring monitoring and management starting in Primary Health Care ^11^ ^,^ ^27^. 

The prevalence of anemia in patients with advanced stages of chronic kidney disease observed in this study was 68.4%. In the literature, the presence and severity of anemia have been associated with increased progression to end-stage chronic kidney disease and the need for supportive therapy ^15^ ^,^ ^28^ ^,^ ^29^. 

In this cohort, the risk of a patient with chronic kidney disease and anemia progressing to the need for renal replacement therapy within five years was 3.24 times higher (95%CI 1.38; 7.61) compared to patients without anemia at the start of outpatient follow-up. The hemoglobin level at the start of specialized follow-up was an independent variable associated with the outcome (HR 0.766; 95%CI 0.593; 0.989).

It is recommended that all patients with chronic kidney disease be monitored in Primary Health Care, regardLess of the stage or etiology ^9^. The primary care physician should order tests such as serum creatinine, electrolytes, and blood count, managing chronic kidney disease and modifiable risk factors for the disease. Additionally, it is recommended that patients in stages 3B, 4, and 5 be followed up by nephrologists ^9^ ^,^ ^30^.

This study had some limitations, such as: 

Retrospective data analysis, given that the data available in the medical records were not controlled or recorded by the research team; therefore, they were not always fully available for collection at the time of patient inclusion and follow-up; 

Patient monitoring for up to five years, which may have been influenced by administrative issues at the center, as well as individual and sociodemographic factors of the patients; 

Lack of data on continuous medication use for 90 patients (16.8%), resulting in analysis of 444 out of the 534 included patients for this variable; and

A small number of patients experienced the studied outcome (22; 4.1% of the total).

There was a high prevalence of anemia among patients with chronic kidney disease admitted to a specialized nephrology outpatient service, and this condition was associated with other dysfunctions, such as a faster decline in estimated glomerular filtration rate, mineral bone disorder, and dyslipidemia, leading to an increased risk of requiring renal replacement therapy. Considering that these patients were admitted through referrals from Primary Health Care, early diagnosis and proper management of chronic kidney disease by the Primary Health Care physician are essential, even before nephrologist follow-up, aiming to reduce disease progression.
