# Supplementary material for: Prevalence of anemia in patients with chronic kidney disease attended at a nephrology outpatient clinic and its association with the outcome of renal replacement therapy over five years: a retrospective cohort study, Municipality of São Paulo, 2010-2018
Source: Epidemiol Serv Saude. 2026 Mar 9;35:20250206. doi: 10.1590/S2237-96222026v35e20250206.en (PMC12991394; doi:10.1590/S2237-96222026v35e20250206.en)
Supplement: Translation

# Translation: Frequência da anemia em pacientes com doença renal crônica admitidos em um ambulatório de nefrologia e sua associação com o desfecho de terapia renal substitutiva em cinco anos: estudo de coorte retrospectivo, município de São Paulo, 2010-2018

**DOI:** 10.1590/S2237-96222026v35e20250206.pt

**Authors:** Andre Kiyoshi Miyahara, Pedro Henrique Moretti Pepato, Júlia Ferreira Rocha, Alexandre Vizzuso de Oliveira, João Vitor Bozza Maia, Vinicius Diniz, Bruno Pellozo Cerqueira, Maria Amelia Aguiar Hazin, Maria Eugenia Fernandes Canziani, Miguel Angelo Goes

**Affiliations:** ¹ Universidade Federal de São Paulo, São Paulo, SP, Brasil

**Keywords:** Insuficiência Renal Crônica, Anemia, Atenção Primária à Saúde, Prevalência, Estudos de Coortes.

## Abstract

**Objetivo:**

Avaliar a prevalência de anemia em pacientes com doença renal crônica e sua associação com a necessidade de terapia renal substitutiva durante cinco anos em um ambulatório especializado.

**Métodos:**

Estudo de coorte retrospectivo que incluiu pacientes admitidos em um ambulatório de nefrologia entre 2010 e 2018 no município de São Paulo. Analisaram-se dados demográficos, clínicos e laboratoriais a partir de frequências e valores absolutos. A anemia foi definida como concentração de hemoglobina <13 g/dL para homens e <12 g/dL para mulheres. Compararam-se os dados clínicos e a necessidade de terapia renal substitutiva ao longo de cinco anos entre pacientes com e sem anemia na admissão. Para as análises estatísticas, utilizaram-se testes qui-quadrado de Pearson e t de Student ou U de Mann-Whitney, curva de Kaplan-Meier com teste de log-rank e análise de sobrevida por regressão de Cox.

**Resultados:**

O estudo incluiu 534 pacientes, sendo 41,8% diagnosticados com anemia na admissão. As principais etiologias da doença renal crônica foram hipertensão (35,2%) e diabetes (27,5%). Evoluíram para terapia renal substitutiva, em cinco anos, 4,1%. A anemia na admissão foi associada ao maior risco do desfecho (*hazard ratio* - HR 3,24; intervalo de confiança de 95% - IC95% 1,38; 7,61). O nível de hemoglobina mostrou-se preditor independente para o desfecho (HR 0,766; IC95% 0,593; 0,989).

**Conclusão:**

Encontrou-se alta prevalência de anemia na doença renal crônica em pacientes provenientes da Atenção Primária. O nível de hemoglobina foi uma variável independente associada ao desfecho.

**Aspectos éticos**

Esta pesquisa respeitou os princípios éticos, obtendo os seguintes dados de aprovação:

Comitê de ética em pesquisa: Universidade Federal de São Paulo

Número do parecer: 3.200.969

Data de aprovação: 15/3/2019

Certificado de apresentação de apreciação ética: 08636819.8.0000.5505/2019

Registro do consentimento livre e esclarecido: Dispensado.

## Introdução

A doença renal crônica é um problema de saúde pública global, caracterizada por alterações morfofuncionais nos rins que persistem por mais de três meses. Essa condição leva a complicações metabólicas e funcionais, podendo resultar em desfechos clínicos adversos, como a necessidade de terapia renal substitutiva, o maior risco cardiovascular, a fragilidade óssea, a anemia e o aumento da mortalidade ^1^. A doença renal crônica é classificada com base na sua etiologia, na taxa de filtração glomerular estimada e na presença de albuminúria ^1^. As principais causas da doença são o diabetes e a hipertensão, o que reforça a importância do manejo preventivo na Atenção Primária. A necessidade de terapia renal substitutiva surge à medida que a doença renal crônica progride. Essa terapia é indicada quando a taxa de filtração glomerular estimada é inferior a 15 ml/min/1,73 m², associada a síndrome urêmica, distúrbios no equilíbrio ácido-base, dificuldade em manter a euvolemia ou alterações na pressão arterial ^1^. A anemia é uma complicação frequente na doença renal crônica, resultante da deficiência de eritropoetina e agravada por fatores como carências nutricionais e inflamação ^2^ ^,^ ^3^ ^,^ ^4^ ^,^ ^5^ ^,^ ^6^. O estado inflamatório associado à doença renal crônica prejudica a ação da eritropoetina, reduz a absorção intestinal de ferro e dificulta a mobilização dos estoques desse mineral, devido ao aumento da produção hepática de hepcidina ^2^ ^,^ ^3^ ^,^ ^4^. A anemia torna-se clinicamente evidente quando a taxa de filtração glomerular estimada cai abaixo de 30 ml/min/1,73 m², intensificando complicações cardiovasculares e contribuindo para o aumento da mortalidade ^6^ ^,^ ^7^.

Este estudo teve como objetivo avaliar a prevalência de anemia em pacientes com doença renal crônica encaminhados da Atenção Primária para o tratamento conservador em uma unidade especializada em nefrologia no município de São Paulo. Buscou-se analisar a associação da anemia com a necessidade de terapia renal substitutiva ao longo de cinco anos. A proposta visou compreender melhor o perfil da anemia associada à doença renal crônica no contexto brasileiro, o que contribui para o preenchimento da lacuna de estudos epidemiológicos sobre essa condição na população do país.

## Métodos

Delineamento e contexto

Tratou-se de estudo de coorte retrospectivo baseado na análise de prontuários médicos de pacientes admitidos no serviço ambulatorial entre 2010 e 2018, em um Serviço Médico Universitário especializado em doença renal crônica da disciplina de nefrologia da Escola Paulista de Medicina/Universidade Federal de São Paulo. O objetivo foi avaliar a presença de anemia na admissão ao serviço e compará-la entre dois grupos: i) anemia e ii) não anemia. Os pacientes foram acompanhados por cinco anos para a análise da necessidade de terapia renal substitutiva. 

Fonte de dados, mensuração e variáveis

Foram coletados dados clínicos e laboratoriais a partir dos sistemas de prontuários físicos e eletrônicos, os quais incluíram informações demográficas (sexo, idade, medidas antropométricas, comorbidades), etiologia e estágio da doença renal crônica, além de medicamentos em uso. Os exames laboratoriais avaliados incluíram hemograma completo, gasometria venosa, perfil lipídico, perfil glicêmico, perfil de ferro, creatinina, ureia, sódio, potássio, fósforo, paratormônio, cálcio total e análise de urina. A anemia foi definida conforme os critérios da Organização Mundial da Saúde: hemoglobina <13,0 g/dL para homens e <12,0 g/dL para mulheres ^8^. 

O diagnóstico de doença renal crônica foi baseado no KDIGO 2024 para doença renal crônica ^1^. Segundo a diretriz, a doença renal crônica é uma alteração morfofuncional por três meses ou mais, podendo ser visualizada pela presença de marcador de dano renal (um ou mais): albuminúria (≥30 mg/g), sedimentos urinários anormais, hematúria persistente, alterações de eletrólitos por anomalias dos túbulos renais, patologias detectadas na histologia, anomalias estruturais detectadas por imagem ou histórico de transplante renal. A função renal <60 ml/min per 1,73 m^2^ com duração maior que três meses é considerada como doença renal crônica, sendo dividida em:

G1 ≥90 ml/min per 1,73 m^2^; G2 60-89 ml/min per 1,73 m^2^; G3a 45-59 ml/min per 1,73 m^2^; G3b 30-44 ml/min per 1,73 m^2^; G4 15-29 ml/min per 1,73 m^2^; G5 <15 ml/min per 1,73 m^2 (^ ^1^.

Participantes

Os critérios de inclusão foram: pacientes diagnosticados com doença renal crônica admitidos entre 2010 e 2018 que possuíam exames laboratoriais completos, incluindo hemograma, ureia, creatinina, sódio e potássio. Os critérios de exclusão foram: idade inferior a 18 anos; anemia de outras etiologias; início de terapia renal substitutiva ou transplante renal em até 90 dias após a inclusão; ausência de diagnóstico adequado de doença renal crônica; e óbito ocorrido nos primeiros 90 dias após a inclusão.

A anemia de outras etiologias foi diagnosticada com base na história clínica e nos exames laboratoriais, como aumento de DHL, teste de Coombs e haptoglobina. Cada paciente foi avaliado por um profissional médico especialista e capacitado. Os pacientes excluídos não foram admitidos no ambulatório, havendo acompanhamento direto em outras especialidades, como hematologia e reumatologia. Dessa forma, nos resultados não foram contabilizados os pacientes de outras especialidades com anemia de outras etiologias e em acompanhamento com a interconsulta da equipe da nefrologia. 

A ausência de diagnóstico adequado de doença renal crônica foi definida como o não atendimento a um ou mais critérios de inclusão estabelecidos.

O diagnóstico etiológico da doença renal crônica foi feito com base na história clínica e na análise de comorbidades, como hipertensão, diabetes e doença renal policística autossômica dominante. A biópsia ficou restrita aos casos com indicação de biópsia renal, como síndrome nefrítica aguda, síndrome nefrótica, insuficiência renal de causa não esclarecida e glomerulonefrite rapidamente progressiva.

Os pacientes foram inicialmente estratificados com base na presença ou ausência de anemia no início do acompanhamento ambulatorial. O desfecho analisado foi a necessidade de terapia renal substitutiva dentro de cinco anos da admissão. Uma segunda análise foi conduzida para identificar variáveis associadas à progressão para terapia renal substitutiva nesse período, além da necessidade do uso de eritropoetina em dois anos após a admissão. 

O tratamento com eritropoetina foi seguido pelos Protocolos Clínicos e Diretrizes Terapêuticas da anemia da doença renal crônica do Ministério da Saúde, indicando seu uso quando hemoglobina <10 g/dL ^9^.

Viés

O delineamento de centro único, embora facilite o controle de variáveis operacionais e logísticas, pode limitar a generalização dos resultados. Isso ocorre porque as características populacionais, as práticas assistenciais e a infraestrutura local podem não refletir a realidade de outros centros, introduzindo viés de seleção e comprometendo a validade externa do estudo.

Tamanho do estudo

O estudo incluiu todos os pacientes admitidos no ambulatório e que apresentavam dados clínicos e laboratoriais completos e adequados aos critérios de inclusão, totalizando 534 pacientes.

Método estatístico

O nível de significância estatística foi considerado p-valor<0,05. As análises estatísticas foram realizadas utilizando o software SPSS Statistics Version 21 (IBM, Armonk, Nova York, Estados Unidos). As variáveis categóricas foram expressas em frequências absolutas e percentuais, sendo comparadas pelo teste qui-quadrado de Pearson. As variáveis quantitativas foram comparadas entre os grupos pelos testes t de Student e U de Mann-Whitney, de acordo com o teste de normalidade Kolmogorov-Smirnov. Realizou-se análise de Kaplan-Meier com teste de log-rank para comparar a sobrevida renal entre os grupos com e sem anemia na admissão. A sobrevida renal foi considerada como ausência de necessidade de terapia renal substitutiva ou transplante renal em cinco anos.

A regressão de Cox foi utilizada para identificar variáveis associadas ao desfecho, com ajuste para potenciais fatores de confusão. As variáveis incluídas no modelo de regressão foram estatisticamente significativas e não apresentaram multicolinearidade pelo diagnóstico de colinearidade por meio do fator de inflação da variância. Na análise, foram incluídos a taxa de filtração glomerular estimada, o colesterol total, a hemoglobina, a idade e a lipoproteína de baixa densidade. 

## Resultados

Na fase inicial do estudo, foram coletados 889 prontuários de pacientes iniciando acompanhamento ambulatorial para doença renal crônica no serviço de nefrologia. Em seguida, foram excluídos 355 pacientes por: serem menores de 18 anos (n=18); terem iniciado a terapia de substituição renal (n=10) ou ido a óbito (n=1) dentro de 90 dias após o início do acompanhamento ambulatorial; e não possuírem exames suficientes para o estudo atual (n=326). 

No estudo, foram incluídos 534 pacientes ambulatoriais (Figura 1), com idade média de 69,4 anos. Desses pacientes, 295 (55,2%) eram do sexo masculino. As principais etiologias da doença renal crônica nos pacientes admitidos foram hipertensão (35,2%) e diabetes (27,5%), seguidas por nefrite tubulointersticial (12,0%), glomerulonefrite crônica (7,0%) e doença renal policística autossômica dominante (4,0%). A primeira análise estratificou os participantes elegíveis pelo status de anemia no início do acompanhamento ambulatorial. Observou-se que 223 (41,8%) apresentavam anemia. 

**Figura 1:** Pacientes com doença renal crônica admitidos no ambulatório especializado em nefrologia e incluídos no estudo. São Paulo, 2010-2018

Estágios iniciais de doença renal crônica foram mais prevalentes no grupo sem anemia do que no grupo com anemia (p-valor<0,001). O uso prévio de ferro e a suplementação de vitamina B12 foram mais frequentes no grupo com anemia. Não houve significância estatística para o uso de agentes hipoglicemiantes, vitamina D e anti-hipertensivos, como inibidores da enzima conversora de angiotensina e bloqueadores dos receptores de angiotensina entre os grupos (Tabela 1). Dos pacientes admitidos com anemia, 13,9% necessitaram de eritropoetina recombinante humana dentro de dois anos, em comparação com 1,6% no grupo sem anemia (p-valor<0,001). 

**Tabela 1.** Comparação entre grupos de pacientes com doença renal crônica admitidos no ambulatório com e sem anemia. São Paulo, 2010-2018 (n=534)

	Sem anemia (n=311)	Anemia (n=223)	
	n (%)	n (%)	p-valor
Sexo masculino	177 (56,9)	118 (52,9)	0,359
Estágio doença renal crônica			<0,001
1	2 (0,6)	0 (0,0)	
2	14 (4,5)	9 (4,0)	
3A	69 (22,2)	13 (5,8)	
3B	136 (43,7)	74 (33,2)	
4	85 (27,3)	110 (49,3)	
5	5 (1,6)	17 (7,6)	
Medicamentos em uso^a^			
Suplementação de ferro	6 (2,4)	18 (9,4)	0,001
Vitamina B12	13 (5,1)	21 (11,00)	0,022
Eritropoetina em dois anos	5 (1,6)	30 (13,9)	<0,001

^a^ Disponíveis apenas para 444 pacientes.

Os pacientes do grupo anemia que iniciaram o acompanhamento ambulatorial apresentaram menor taxa de filtração glomerular estimada, bicarbonato sérico, concentração de hemoglobina, hematócrito, concentração média de hemoglobina corpuscular e níveis de ferro sérico. Em contrapartida, os pacientes desse grupo apresentaram maior RDW, potássio, fósforo e paratormônio intacto (Tabela 2).

**Tabela 2.** Comparação entre grupos de pacientes com doença renal crônica admitidos no ambulatório com e sem anemia. São Paulo, 2010-2018 (n=534)

	Sem anemia (n=311)	Anemia (n=223)	p-valor
	Mediana (IIQ)	Mediana (IIQ)	
Índice de massa corpórea (kg/m^2^)	27,2 (23,9-31,1)	26,3 (23,9-29,7)	0,251^a^
Taxa de filtração glomerular estimada (ml/min/1,73m²)	35,6 (28,5-45,7)	27,5 (20,3-36,9)	<0,001^a^
Hemoglobina (g/dL)	13,9 (13,1-14,9)	11,2 (10,4-11,8)	<0,001
RDW ^b^ (%)	11,8 (11,3-12,6)	12,3 (11,3-13,2)	0,012^a^
Hemoglobina corpuscular média (pg)	30,1 (28,5-31,1)	29,5 (27,8-30,9)	0,037^a^
Gasometria venosa pH ^c^	7,31 (7,27-7,34)	7,30 (7,25-7,34)	0,064^a^
Bicarbonato (mEq/l)	26,6 (24,1-29,1)	25,4 (22,0-27,8)	<0,001
Ferro (mcg/dL)	79,5 (67,0-100,2)	65,5 (50,5-88,2)	0,002
Ferritina (ng/ml)	138,5 (75,1-254,3)	188,5 (91,0-299,5)	0,072^a^
Saturação transferrina (%)	27,0 (22,0-35,0)	25,0 (18,5-33,4)	0,328
Fósforo (mg/dL)	3,4 (3,0-3,8)	3,8 (3,5-4,3)	<0,001
Sódio (mEq/l)	139 (138-141)	139 (138-142)	0,637^a^
Potássio (mEq/l)	4,7 (4,4-5,0)	4,9 (4,5-5,3)	0,001
PTH (pg/ml) ^d^	99,5 (62,7-156,7)	132,0 (82,0-213,0)	0,001^a^
Colesterol total (mg/dL)	181 (155-210)	175 (143-207)	0,414

^a^ Variáveis com distribuição não normal; ^b^ RDW: *red cell distribution width*; ^c^ pH: potencial hidrogênio iônico; ^d^ PTH: paratormônio.

Na segunda análise, os pacientes foram estratificados com base no desfecho de necessidade de terapia renal substitutiva em cinco anos após o início do acompanhamento ambulatorial. Compararam-se os dados clínicos de linha de base entre esses grupos. Dos 534 pacientes do estudo, 22 (4,1%) progrediram para terapia renal substitutiva em cinco anos, e todos foram encaminhados para um programa de hemodiálise. Os pacientes que foram para diálise eram mais jovens (54,5 [48,0-67,3]; 65,0 [56,0-73,8] anos; p-valor 0,030), apresentavam menor taxa de filtração glomerular estimada (27,0 [15,4-32,0]; 32,6 [25,1-42,2] ml/min per 1,73 m^2^; p-valor 0,011), níveis mais elevados de colesterol total (194,5 [161,8-243,5]; 179,0 [151,0-207,8] mg/dL; p-valor 0,004) e de lipoproteína de baixa densidade (120,5 [92,8-154,0]; 102,0 [80,0-127,0] mg/dL; p-valor 0,004), além de concentrações menores de hemoglobina (11,9 [10,5-12,9]; 12,8 [11,4-14,0] g/dL; p-valor 0,032) na admissão (Tabela 3). 

**Tabela 3.** Comparação entre pacientes que progrediram para a terapia renal substitutiva dentro de cinco anos e aqueles que não necessitaram. São Paulo, 2010-2018 (n=534)

Variável	Não diálise (n=512)	Diálise (n=22)	p-valor
	n (%)	n (%)	
Masculino	280 (54,7)	15 (68,2)	0,213
Anemia na admissão	208 (40,6)	15 (68,2)	0,011
Etiologia doença renal crônica			0,012
Diabetes	137 (26,8)	10 (45,4)	
Hipertensão	185 (36,1)	3 (13,6)	
Glomerulonefrite crônica	34 (6,6)	5 (22,7)	
Nefrite intersticial crônica	64 (12,5)	2 (9,1)	
Indeterminado	70 (13,7)	2 (9,1)	
Doença renal policística autossômica dominante	22 (4,3)	0 (0,0)	
Medicamentos da admissão^a^			
Inibidor da enzima conversora de angiotensina	155 (36,6)	6 (28,6)	0,457
Bloqueador do receptor de angiotensina	117 (27,6)	9 (42,9)	0,13
Diuréticos de alça	165 (38,9)	14 (66,7)	0,011
Bloqueador do canal de cálcio	148 (34,9)	12 (57,1)	0,038
Suplementação de ferro	20 (4,7)	4 (19,1)	0,005
Uso de eritropoetina em dois anos	28 (5,5)	8 (36,4)	<0,001

^a^ Disponíveis apenas para 444 pacientes.

No grupo diálise, foram observadas maiores prevalência de diabetes como etiologia da doença renal crônica (45,4; 26,8%; p-valor 0,012) e frequência de anemia (68,2; 40,6%; p-valor 0,011). Dos 22 pacientes que necessitaram de diálise, 36,4% utilizaram eritropoetina em algum momento durante os dois primeiros anos de acompanhamento, em comparação com 5,5% daqueles que não progrediram para terapia renal substitutiva (p-valor<0,001). Os pacientes que necessitaram de terapia renal substitutiva tinham maior uso de diuréticos de alça, bloqueadores dos canais de cálcio diidropiridínicos e suplementação de ferro (Tabela 3). 

A curva de Kaplan-Meier demonstrou que pacientes com doença renal crônica e anemia apresentaram maior necessidade de hemodiálise em cinco anos de acompanhamento (*hazard ratio -* HR 3,24; intervalo de confiança de 95% - IC95% 1,38; 7,61; p-valor<0,001), havendo 44 pacientes com perda de seguimento (Figura 2). Na análise de regressão de Cox, a taxa de filtração glomerular estimada (HR 0,977; IC95% 0,940; 1,017; p-valor 0,259), os níveis de colesterol total (HR 1,009; IC95% 0,995; 1,024; p-valor 0,211) e a lipoproteína de baixa densidade (HR 1,004; IC95% 0,986; 1,023; p-valor 0,643) não apresentaram significância estatística. A idade (HR 0,973; IC95% 0,948; 0,998; p-valor 0,036) e a hemoglobina (HR 0,766; IC95% 0,593; 0,989; p-valor 0,041) foram identificadas como preditores independentes significativos no desfecho.

**Figura 2.** *Hazard ratio* (HR) e intervalo de confiança de 95% (IC95%) para a necessidade de terapia renal substitutiva dentro de cinco anos de acompanhamento de pacientes com e sem anemia. São Paulo, 2010-2018 (n=534)

No grupo anemia, houve um paciente que faleceu (0,4%) dentro de cinco anos após o início do acompanhamento. Esse óbito ocorreu após três anos de inclusão no serviço ambulatorial. Nenhum óbito foi registrado no grupo sem anemia em cinco anos de acompanhamento. 

## Discussão

O principal achado deste estudo foi a presença frequente de anemia em pacientes com doença renal crônica que iniciam o acompanhamento em um serviço ambulatorial especializado pelo Sistema Único de Saúde brasileiro. Identificou-se que 41,8% dos pacientes ambulatoriais inscritos apresentaram anemia na admissão. Esses pacientes foram encaminhados pelas unidades básicas de saúde por meio do Centro Regulador de Oferta de Serviços de Saúde do Estado de São Paulo, que direciona os pacientes encaminhados com base no prognóstico e na urgência para acesso ao serviço, considerando os horários disponíveis e a divisão territorial.

Essa taxa alarmante de anemia não é apenas significativa, mas também supera em muito a prevalência observada em populações com doença renal crônica em outros países, onde a anemia é frequentemente identificada nos estágios iniciais do processo da doença ^2^ ^,^ ^10^ ^,^ ^11^ ^,^ ^12^. Avaliações abrangentes e monitoramento preventivo são normalmente realizados apenas nos estágios mais avançados da doença renal crônica, o que é preocupante, considerando os efeitos bem documentados da anemia sobre os desfechos de saúde em pacientes com doença renal crônica.

A anemia na doença renal crônica resulta principalmente da deficiência de eritropoetina e é influenciada por outros fatores que se agravam à medida que a doença progride. A produção reduzida de eritropoetina, combinada com deficiências vitamínicas, inflamação e metabolismo de ferro desregulado, prejudica a produção de glóbulos vermelhos. Infecções e acúmulo de solutos urêmicos podem exacerbar a resposta inflamatória ou resultar de comorbidades e condições subjacentes de saúde ^2^ ^,^ ^3^ ^,^ ^4^ ^,^ ^5^ ^,^ ^13^ ^,^ ^14^.

A queda da concentração de hemoglobina afetou negativamente a progressão da doença renal crônica em 265 pacientes com diabetes tipo 2 (HR 0,65; IC95% 0,48; 0,88; p-valor 0,005). Níveis mais baixos de hemoglobina (<13,3 g/dL) foram preditor independente de dano renal ^15^. A presença de anemia está associada a valores mais baixos de taxa de filtração glomerular estimada, independentemente da relação causa-efeito ^4^ ^,^ ^5^ ^,^ ^6^ ^,^ ^12^ ^,^ ^13^. Este estudo converge com os achados da literatura internacional, onde há maior prevalência de anemia nos estágios avançados da doença renal crônica, sugerindo a solidez do conhecimento médico-científico sobre essa condição ^16^ ^,^ ^17^. Mesmo com a literatura bem estabelecida sobre as consequências da anemia renal, ainda foram encontrados pacientes com doença renal crônica e anemia sem intervenção terapêutica apropriada, especialmente entre pacientes não dialíticos com doença renal crônica.

A presença de anemia tem sido associada a uma taxa mais rápida de progressão da doença renal crônica ^18^. Portanto, isso deve ser um dos focos do reconhecimento e acompanhamento da doença. O prognóstico ruim do paciente ambulatorial e os desfechos adversos podem estar associados à presença concomitante de anemia e à redução da taxa de filtração glomerular estimada, como eventos cardiovasculares isquêmicos ou necessidade de terapia renal substitutiva ^19^ ^,^ ^20^. Esses desfechos têm considerável morbidade e mortalidade e exigem intervenção médica com, consequentemente, maiores custos para centros públicos e privados. Com base neste estudo e na literatura estabelecida, os resultados sugerem que os médicos generalistas podem monitorar a anemia desde os estágios iniciais da doença renal crônica para mitigar a progressão para terapia renal substitutiva, reduzir a morbimortalidade e diminuir os custos por paciente.

A presença de anemia também foi associada a níveis séricos mais elevados de fósforo e PTH (paratormônio), fatores relacionados ao distúrbio mineral ósseo - comum nos estágios avançados da doença renal crônica -, condição associada às maiores morbidade e mortalidade nessa doença ^21^. O grupo de pacientes com anemia apresentou valores mais baixos de bicarbonato, associados à tendência de menores valores de pH na gasometria venosa, que ocorre devido a alterações na produção renal e reabsorção de íons bicarbonato e excreção de íons hidrogênio ^22^. Estima-se que a acidose metabólica esteja presente em cerca de 15,0% dos pacientes com doença renal crônica, com um risco 40,0% maior em pacientes anêmicos com doença renal crônica ^23^. Priorizar o manejo da anemia é essencial para melhorar a saúde geral e reduzir complicações em pacientes com doença renal crônica.

Foram feitas observações sobre a necessidade de terapia renal substitutiva em cinco anos após o início do acompanhamento ambulatorial especializado em doença renal crônica. Ocorreu maior frequência de etiologia de doença renal crônica por diabetes (45,5%) do que por hipertensão (13,4%) nos pacientes que progrediram para terapia renal substitutiva. Esse achado difere dos dados da literatura brasileira, que relatam que 33,0% dos casos de doença renal terminal foram atribuídos à hipertensão e 32%, ao diabetes ^24^. No entanto, são consistentes com achados de outros países, onde o diabetes é a principal causa de doença renal crônica que progride com necessidade de terapia renal substitutiva ^25^ ^,^ ^26^. Os pacientes em diálise também apresentaram mais alterações no perfil lipídico, com níveis mais elevados de colesterol total e lipoproteína de baixa densidade. Pacientes dislipidêmicos tendem a progredir para doença renal crônica com pior prognóstico e risco cardiovascular significativo, exigindo acompanhamento e manejo desde a Atenção Primária ^11^ ^,^ ^27^. 

A prevalência de anemia em pacientes com doença renal crônica em estágios avançados observada neste estudo foi 68,4%. Na literatura, a presença e a gravidade da anemia têm sido associadas à maior progressão para doença renal crônica terminal e necessidade de terapia de suporte ^15^ ^,^ ^28^ ^,^ ^29^. 

Nesta coorte, o risco de um paciente com doença renal crônica e anemia desenvolver a necessidade de terapia renal substitutiva em cinco anos foi 3,24 (IC95% 1,38; 7,61) vezes maior do que os pacientes sem anemia desde o início do acompanhamento ambulatorial. O nível de hemoglobina no início do acompanhamento especializado foi uma variável independente associada ao desfecho (HR 0,766; IC95% 0,593; 0,989).

Recomenda-se que todos os pacientes com doença renal crônica sejam monitorados na Atenção Primária à Saúde, independentemente do estágio ou da etiologia ^9^. O médico da Atenção Primária deve solicitar exames como creatinina sérica, eletrólitos e hemograma, manejando a doença renal crônica e os fatores de risco modificáveis da doença. Além disso, recomenda-se que pacientes nos estágios 3B, 4 e 5 sejam acompanhados por nefrologistas ^9^ ^,^ ^30^.

Este estudo possuiu algumas limitações, como: 

análise de dados retrospectiva, visto que os dados disponíveis nos prontuários médicos não foram controlados e descritos pela equipe de pesquisa; portanto, nem sempre estavam totalmente disponíveis para coleta nos momentos da inclusão e do acompanhamento do paciente; 

monitoramento de pacientes por até cinco anos, o que pode ter sido influenciado por questões administrativas no centro, bem como fatores individuais e sociodemográficos dos pacientes; 

falta de dados sobre medicamentos em uso contínuo de 90 pacientes (16,8%), resultando na análise de 444 dos 534 pacientes incluídos para essa variável; e

número reduzido de pacientes que apresentaram o desfecho estudado (22; 4,1% do total).

Houve alta frequência de anemia em pacientes com doença renal crônica admitidos em um serviço ambulatorial especializado em nefrologia, e essa condição se correlaciona com outras disfunções, como maior velocidade de redução da taxa de filtração glomerular, distúrbio mineral ósseo e dislipidemia, com maior risco de necessidade de terapia renal substitutiva. Considerando que esses pacientes foram admitidos por meio de encaminhamentos da Atenção Primária, são necessários o diagnóstico precoce e o manejo adequado da doença renal crônica pelo médico da Atenção Primária, mesmo antes do acompanhamento por nefrologistas, visando reduzir a progressão da doença.
